# Slow Sand Filters for the 21st Century: A Review

**DOI:** 10.3390/ijerph20021019

**Published:** 2023-01-05

**Authors:** John K. Maiyo, Sruthi Dasika, Chad T. Jafvert

**Affiliations:** 1 Division of Environmental and Ecological Engineering, Purdue University, 500 Central Drive, West Lafayette, IN 47907, USA; 2 Lyles School of Civil Engineering, Division of Environmental and Ecological Engineering, Purdue University, 550 Stadium Mall Drive, West Lafayette, IN 47907, USA

**Keywords:** biosand filter, contaminant removal, filter design, filter media, water quality, water treatment

## Abstract

Safe drinking water remains a major global challenge, especially in rural areas where, according to UNICEF, 80% of those without access to improved water systems reside. While water, sanitation, and hygiene (WASH)-related diseases and deaths are common outcomes of unsafe water, there is also an economic burden associated with unsafe water. These burdens are most prominent in rural areas in less-developed nations. Slow sand filters (SSFs), or biological sand filters (BSFs), are ideal water treatment solutions for these low-resource regions. SSFs are the oldest municipal drinking water treatment systems and improve water quality by removing suspended particles, dissolved organic chemicals, and other contaminants, effectively reducing turbidity and associated taste and odor problems. The removal of turbidity and dissolved organic compounds from the water enables the use of low-cost disinfection methods, such as chlorination. While the working principles of slow sand filtration have remained the same for over two centuries, the design, sizes, and application of slow sand filters have been customized over the years. This paper reviews these adaptations and recent reports on performance regarding contaminant removal. We specifically address the removal of turbidity and microbial contaminants, which are of great concern to rural populations in developing countries.

## 1. Introduction

In 2017, approximately 30% of the world population still lacked access to a safely managed drinking water source, with 159 million people collecting drinking water directly from surface water sources [1]. Safe drinking water access is a primary issue in rural communities of developing nations, with UNICEF reporting that 80% of people without access to an improved drinking water source live in rural areas [1,2]. Sub-Saharan Africa still lags behind with only 27% access to safely managed water systems, and a further 34% access to a basic water supply [1,3]. As a result of limited access to safe water, the disease burden is worse in developing regions. In 2012, over half a million deaths in low- and middle-income countries were attributed to drinking water [4]. These WASH-related diseases can be controlled with adequate access to safe water, proper sanitation, improved hygiene, and better water management practices, thus preventing around 10% of the global disease burden [5,6].

For the predominantly rural regions in need, safe water technologies must be effective, and slow sand filtration (SSF) has distinguished itself as a suitable water treatment technology for such rural areas [7]. Often used in conjunction with a secondary treatment process, SSFs are effective at reducing (though not necessarily removing completely when applied alone) waterborne pathogens and turbidity [8,9], such that inexpensive alternatives to boiling can be used for final disinfection. Over half a million persons in developing nations currently use SSFs to treat their drinking water [10]. This paper reviewed the traditional SSF design and subsequent design adaptation; the characteristics and their effect on SSF performance; the SSF operating conditions and their effects on SSF performance; and SSFs’ contaminant removal efficiencies. We limited our scope to drinking water applications, and thus wastewater and water reuse applications of SSF were not covered. This review prioritized the removal of turbidity and microbial contaminants, as these are the water quality parameters of concern in rural or under-resourced settings.

Early reported uses of SSFs occurred at John Gibb’s bleachery in Paisley in 1804, in Greenock in 1827, at the Chelsea Water Company in 1829, at the Gorbals Sanitation and Water Company in 1846, and in cholera-stricken London in 1852 [8,11,12,13]. After John Snow linked cholera and typhoid to water contamination, SSF use was mandated by law for all water sourced from the River Thames after 1892 [8,14]. In the early 1900s, SSFs ceded their popularity to chemical treatment (i.e., with flocculation, coagulation, and settling) followed by rapid sand filtration, which requires less space and can be performed on water with more significant variation in source water quality [12]. In the 1980s, however, the demand for smaller water systems rekindled the interest in SSF. The simplicity of their design and their lower energy requirements make SSFs ideal for smaller water treatment facilities [12]. Other advantages include ease of operation and the ability to construct them locally, using locally sourced materials and without requiring specialized equipment [8].

SSFs improve water quality by removing particles, dissolved organic chemicals, and other contaminants, thus reducing turbidity and associated taste and odor problems. Suspended organic materials in water are filtered by the sand and are then slowly mineralized by microorganisms in the filter’s biofilms. Since organic removal occurs through (predominantly aerobic) biological processes, a slow sand filter’s hydraulic residence time (HRT) is the key design parameter controlling the effluent water quality. In most technical reviews of slow sand filters, there is a general lack of appreciation for the HRT as the key design parameter. The HRT (h) is a function of the flow rate (Q, m^3^/h), total volume of the sand (V, m^3^), and sand porosity (*n*), and is mathematically defined by:HRT = V·*n*/Q.

The porosity of sand is generally in the range of 0.35 to 0.5, such that 35 to 50% of the active filter’s volume is occupied by water in contact with microorganisms attached to the sand grains. To have a high surface area while also maintaining a high porosity, the sand should be of reasonably uniform size, as a wide range of particle sizes will decrease the porosity, which in turn will decrease the HRT.

Therefore, the two parameters that best describe an SSF for design and comparison purposes are the hydraulic retention time and sand grain size distribution (i.e., sand surface area). Clearly, local water quality parameters (including temperature) also determine filter performance and may require adjustments in relation to these two physical parameters. For filters installed in Colombia, Tanzania, Kenya, China, and Thailand by the authors, the sand is sieved at 0.35 mm and 1.5 mm (to remove fine and coarse sand), providing excellent treatment at an HRT of 8 to 12 h. Below, each of these design concepts is described in more detail.

## 2. Mechanisms of Pollutant Removal

As the water flows by gravity through the sand, contaminants are removed by both physical and biological processes. The sand medium provides a large surface area for non-pathogenic aerobic microorganisms to attach, which metabolize any organic matter that enters the filter in the influent water. These microorganisms also prey on bacteria and viruses that enter the filter in the influent water. At the same time, physical mechanisms (inertial impaction and attachment, diffusion, and adsorption) collect or effectively strain particles onto the sand surfaces [15]. If these particles are composed of organic matter, they will further undergo biological decay. Particle removal occurs predominately at the top of the sand bed, in the surface layer known as the Schmutzdecke or biofilm layer [8,16]. Dissolved organic matter that enters the filter, or that is created from particle decay in the Schmutzdecke layer, can be mineralized lower in the filter.

The pore space between the sand particles is always completely saturated with water, with a layer of water always present above the sand and exposed to atmospheric molecular oxygen. Hence, after weeks to months of operation, the Schmutzdecke (biofilm) layer generally forms on and within the first 2 to 5 cm of the sand, due to the higher removal rate of suspended particles within this layer. Up to 60% bacteria removal [17] and 0.56 log_10_ MS2 reduction per cm [18] has been reported within the top 5 cm of the filter medium, whereas only 0.06 log_10_ MS2 reduction per cm has been reported through the remainder of the filter [18]. The disproportionately high removal of microorganisms at the top of the filter medium is consistent with a more diverse microbial population within this zone [18]. The removal efficiency of the top biofilm layer is dependent on the state of development (i.e., maturity) of the biofilm. The disruption of the biofilm during filter cleaning, for instance, was shown to result in a decline in bacteria removal by 1–2 log_10_ [19]. In another study, it was demonstrated that the development of the biofilm increased coliform removal by an order of magnitude, yet had no significant effect on Giardia removal [20]. Biological activity in the layer has been shown or suggested to enhance the removal of bacteria [19], viruses [21], phages [18], and organic contaminants. Physical-chemical processes occurring in the top biofilm layer include screening, sedimentation, interception, diffusion, absorption, and flocculation. In contrast, the biological processes include predation, natural death, the scavenging of detritus, inactivation, bio-antagonism, and metabolic decay and mineralization [8,12,14,22,23]. Screening occurs when particles larger than the pore sizes of the medium are trapped as the water moves through the filter. Since particle deposition leads to progressively decreasing filter pore size, the removal efficiency of screening increases with time and the maturity of the biofilm [8,24]. Sedimentation occurs when those particles that are denser than water “settle” onto the filter medium, with the result being the same as that of particle screening, due to the reduction in the filter pore size. This is most significant for larger particles over 4 μm in diameter [22].

Particles that are less than 1 μm in diameter are affected by Brownian motion, leading to particle diffusion, which may cause contact with and attachment to the sand surface. The particles attached to or deposited on the medium can also be detached due to changes in flow or other disturbances. Particle detachment leads to penetration deeper into the filter and potentially particle breakthrough [8,22,24]. Adsorption generally refers to the attachment of dissolved chemicals to the medium.

Predation has been proposed as the primary process resulting in the removal and inactivation of microbial pathogens in slow sand filters [8]. Larger microorganisms, such as protozoa, feed on smaller microorganisms and particles, such as bacteria and viruses [21]. Elliot et al. (2011) concluded that the activity of the microbial community within household filters was responsible for the MS2 and PRD-1 reductions in their study. They hypothesized that the production of microbial exoproducts, such as proteolytic enzymes, and grazing on viruses were possible pathways leading to inactivation [21]. Poynter and Slade (1977) also concluded that the removal of bacteria and viruses was primarily achieved through biological processes. The authors inferred this conclusion from the effects of temperature, flow regimes, and filter maturation on filtration efficiency [15]. By using stable isotope metagenomics, a lab-scale SSF study preformed in Glasgow identified and attributed the attrition of *E. coli* to several protozoan species. Bacterial antagonism can refer to the inactivation of pathogenic microorganisms by other organisms through simply out-competing the pathogens with respect to nutrient uptake and growth, essentially resulting in pathogen attrition. The presence of naturally occurring bacteria has been reported to decrease pathogens in filters [8,24].

Regarding the mineralization of organic matter within the biofilm, bacteria are particularly efficient at metabolizing natural organic contaminants. Bai (2013) reported that the biofilm was dominated by bacteria (up to 90% compared to other microorganisms in the biofilm) and that these microbes were significant in the breakdown of aromatic compounds, nitrogen removal, and even the (temporary) removal of heavy metals (Mn^2+^ and As^3+^) from groundwater [25]. Importantly, a slow sand filter is primarily an aerobic treatment technology, and the water that enters the filter should be close to saturation with respect to molecular oxygen (O_2_); otherwise, the filter can become anaerobic due to the mineralization of organic matter in the filter, resulting in foul-smelling water. This is also why the supernatant water layer above the filter must always be open to the atmosphere, as the biofilm layer that develops on the top of each filter has a large oxygen demand, and the mass transport of O_2_ to this layer should not be restricted.

## 3. Filter Characteristics

The performance of slow sand filters can be quantified by several measurable parameters, including: (1) reduction in water turbidity (i.e., particle removal); (2) reduction in water color; (3) reduction in total organic carbon (TOC) concentration; (4) reduction in pathogen concentration; and (5) reduction in specific organic chemical pollutant concentrations of regional concern. The performance of slow sand filters depends on several factors. As stated above, the removal efficiency of any of these parameters is influenced by the physical and operational characteristics of the filter, with hydraulic retention time, sand particle size, and temperature being the most important. Additionally, for any filter operated at a given hydraulic retention time, temperature, and sand size, the final effluent water quality is also affected by the filter’s biological maturity and feed water quality [8].

### 3.1. Filter Design

The design of each SSF influences not only the ease of installation and operation, but also filter performance. Slow sand filters may be designed and constructed for individual households (i.e., 1–10 people); for schools or small communities (i.e., 10–1000 people); or for small municipalities (i.e., 1000–5000 people). Because water treatment with SSFs is basically a biological process (affected by temperature) and is always applied to a local water source with unique characteristics, it is always advisable to locally construct a small unit (i.e., 20–50 L) to determine the appropriate hydraulic retention time to be used in designing any larger filters. Importantly, even in tropical climates, there may be seasonal differences in local water quality that may affect treatment, including water turbidity due to seasonal rains and the amount and recalcitrance of organic carbon due to seasonal vegetation dynamics, including changes due to local agricultural practices. Knowing the daily water demand for the household or community (Q, L/day) and the necessary hydraulic retention time (HRT, days) allows for the simple calculation of the volume of sand (L) required within the filter(s), V = HRT·Q/*n*, where it may be assumed that the porosity of the sand is approximately *n* = 0.4. Once the volume of sand is calculated (i.e., scale), the filter(s) may be designed using various geometries; construction materials (i.e., medium, basin, fittings); and modes of operation (batch or continuous). Table 1 provides some typical characteristics of continuous-flow slow sand filters that are consistent with the numbers provided above regarding typical hydraulic retention times.

#### Filter Design Modifications

**Figure 1:**
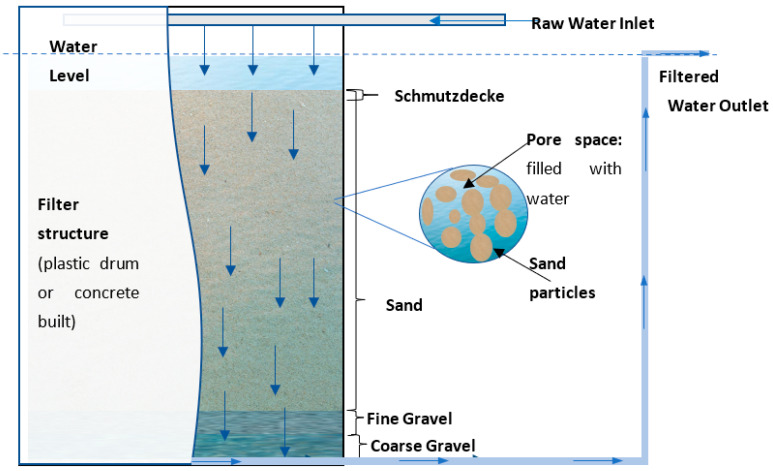
Schematic of a general filter design.

As shown in Figure 1 (the schematic of a general filter design), traditional SSFs have four discrete layers: a coarse gravel underdrain, a finer gravel layer, a larger sand layer, and the supernatant water [8,22]. The underdrainage layer provides support for the finer gravel and sand layers and ensures the uniform downward flow of water in the overlying layers before the abstraction of the treated water. The finer gravel layer prevents the sand from entering the coarse gravel layer. The supernatant water layer provides the necessary pressure head to force water flow through the porous medium layers [12], with the flow rate adjusted by varying the head difference between the supernatant water and the height at which the effluent pipe is open to the atmosphere.

Dr. Manz showed that operating filters intermittently provided good treatment [27]. These “batch” filters, often referred to as biosand filters (BSFs) to differentiate them from SSFs, are designed for use in small communities, schools, and households. Intermittent operation introduces a pause period; that is, a period of time when the water remains in the filter between water changes. The length of the pause time is a compromise between a sufficient HRT for adequate treatment and the frequent supply of nutrients to sustain the microbial community [27,31]. The “water demand” often also determines the pause period.

Biosand filters have been designed and implemented around the world. Concrete biosand filters designed by CAWST (Centre for Affordable Water and Sanitation Technology) in Calgary, Canada, for instance, have been constructed or distribute by at least 450 organizations in over 55 countries [27,32]. Additionally, over the past two decades, the significant distribution of biosand filters made of plastic, such as the 60 L HydrAid filter—distributed by Triple Quest, of Grand Rapids USA, has occurred [33,34]. Plastic BSFs are less expensive to construct and lighter, by 85% and 80%, respectively, than concrete BSFs [35]. A Cambodian study reported that plastic BSFs improved drinking water quality and reduced diarrheal disease incidence by 59%. The study further claimed that 75−90% of the studied households had paid USD 10 for the plastic BSFs [36].

Purdue University researchers (this paper’s authors) have further adapted the plastic BSF design. The novel filter design replaces the gravel layers with a thin manufactured plastic porous plate, placed within a plastic mesh bag. This replacement reduces the required filter medium to just sand and increases the total pore volume within the active treatment zone in each unit. The use of the porous plate lowers the cost and labor required for installation and maintenance. The porous plates and other small components and tools are easy to ship, such that the only locally sourced materials are the sand and plastic drums. These BSFs are operated in batch and are made from 55-gallon drums that can each treat 200 L/day at an HRT of 8 h. Similar household-scale units are made from standard 5-gallon pails, with a two-pail stacked unit able to treat 30 L/day.

Other modifications have also been made to BSFs to adapt them to specific treatment objectives. In Vietnam, a household BSF was modified for the removal of As, Fe, and Mn from source groundwater prior to drinking [37]. Kanchan^TM^ Arsenic Filters (KAF), modified by adding a top layer containing brick chips and iron nails to plastic bucket filters, achieved 85% to 90% arsenic removal, 90% to 95% iron removal, 85% to 99% total coliform removal, and 80% to 95% turbidity removal. KAF was developed- and distributed in Nepal—by researchers at Massachusetts Institute of Technology of USA, Environment and Public Health Organization of Nepal, and Rural Water Supply and Sanitation Support Programme of Nepal. With over 5000 KAF units deployed, 83% were reported to be in-use after one year. The acceptance, in Nepal, was attributed to the improved appearance, taste, and odor of the filtered water [38].

### 3.2. Filter Media

Various materials have been used as SSF media; however, the most frequently used medium is sand. The medium characteristics that affect filter performance include the effective size (D10), uniformity coefficient (Cu), medium composition, and medium cleanliness. The medium can be characterized by its effective size [22], with the recommended range being from 0.15 mm to 0.4 mm [8,14,22]. Note that the effective particle size (or D10) is defined as the particle size where 90% of the actual particles are larger by total mass weight and, hence, 10% of the total mass weight consists of finer particles. In reviewing many papers, it is often problematic to compare results, as some papers refer to effective size, while others refer to simply particle size, with some ambiguity. Whatever the case, a reduction in sand grain size from 0.62 to 0.13 mm resulted in an increase in coliform removal from 96.0% to 99.4%; however, the same size reduction had no significant effect on Giardia cyst removal [20,39]. A reduction in the effective sand size (D10) from 0.52 to 0.17 mm increased the removal of indicator bacteria by between 0.16 and 0.30 logs [40]. These studies support the conclusion made by Logan et al. that sand grain size is the most significant variable affecting the removal of cryptosporidium cysts and, potentially, other pathogens in SSFs [41].

The uniformity coefficient (Cu) is the ratio of the sieve size (mm) through which 60% by weight of the material passes (D60) to the sieve size (mm) that allows 10% of the material to pass (D10). Sand with a lower uniformity coefficient (i.e., higher uniformity) is preferred, with a maximum recommended value of 3.0 [14]. This is because a more uniform sand grain size results in a greater void volume (i.e., porosity), effectively increasing the HRT at a given flow rate and total filter volume. Only 48% cryptosporidium removal was reported in a slow sand filter using sand with a uniformity coefficient of 3.5 to 3.8 [23]; in contrast, another study using the same river water reported over 99% cryptosporidium removal using sand with a uniformity coefficient of 1.72 [42]. Clearly, the use of a uniform (small) sized medium improves the performance of filters for the removal of cryptosporidium.

Rinsing the sand before commissioning a new filter is also important. A study reported that using prewashed sand lowered the effluent turbidity during the commissioning period [42]. In comparison, a lab study using “contaminated” sand (i.e., sand that had been soaked in wastewater effluent for three days) resulted in lower bacteria removal than when clean sand was used. However, field biosand filters containing contaminated sand (i.e., sand sourced from a river bank that was dried and rinsed but not heated to destroy organics) still attained 95% bacterial removal in Madagascar [43]. In general, sand rinsed of most organic matter and clay material content is recommended; pre-rinsing the sand two or three times with agitation is recommended.

Studies on the effect of using several different layers of media within a filter (in addition to one layer of sand and any additional layers of gravel, typically used for water collection at the bottom of each filter) have yielded mixed results. One study reported that using a single layer of river sand resulted in removal rates comparable to those obtained when using two layers of crushed sand [40]. Another study comparing a stratified filter comprised of layers containing combinations of bauxite residue, zeolite, fly ash, granular activated carbon, and sand to a conventional SSF reported that the stratified filter had significant clogging issues. The stratified filter, however, achieved 97% aluminum removal, 71% TOC removal, and 88% ammonia removal during operation [44].

#### 3.2.1. Media Amendments

Researchers have studied filter performance upon the addition of amendments within, above, or below the sand layer. These amendments include iron filings, zerovalent iron, zeolites, chipped copper, bentonite, and bauxite. Improvements in removal efficiency when amending with iron are dependent on the source water quality, the target contaminant, and the composition of the iron amendment [34]. A study that examined filters containing a 10 cm thick iron-oxide-coated sand layer reported the improved removal of bacteria (increasing from 90% to 99%) when compared to unmodified sand. However, this additional layer did not have any significant effect on turbidity removal [45]. Bradley et al. (2011) reported that concrete biofilters with zerovalent iron amendments achieved a higher reduction in MS2 (7 log_10_ removal) when compared to filters amended with steel wool (over 5 log_10_ removal) or unamended sand (2 log_10_ removal) [34]. In a separate study, fecal streptococci removal was improved (99% vs. 97.2% reduction) by the addition of zerovalent iron [46]. A lab study also showed that amending with iron achieved higher virus removal rates (5 log_10_ vs. 0.5 log_10_) and postulated that adsorption could play a role in the improved removal [34].

The type of amendment is obviously important, as different augmentations provide different filter properties. Mixing a 3 mm layer of zeolite into a 10 cm layer of sand within an overall 25 L bucket filter resulted in an improved reduction in effluent turbidity (80% vs. 93%) and fecal coliform (2.2–3.7 log_10_ vs. 2.7–4 log _10_). However, there was no significant change in *E. coli* removal (1.3–3.7 log_10_ vs. 1.6–3.7 log_10_) [47]. Similarly, a Kenyan study concluded that sand amendment with activated bamboo charcoal, diatomite, bone char, and iron oxide resulted in fluoride removal rates of 90, 85, 81 and 70%, respectively [48]. An Iranian study reported that the average turbidity removal efficiency was consistent (at 99%) for conventional sand filters (SSFs), blast furnace slag-modified filters (SMFs), and zeolite-modified filters (ZMFs). The average *E. coli* reductions (9.99%, 11.02%, and 10.73%) and total hardness reductions (21%, 52%, and 66%) were also similar between SSFs, SMFs, and ZMFs, respectively [49]. In fact, by adding BFS to slow sand filters, the pH and alkalinity of the effluent can both be expected to increase, potentially to the point at which the microbial community within the filter becomes stressed.

Hyde et at. (2013) concluded that copper addition significantly impacted the effluent coliform concentration when water was added to the filter in batch daily (20 L each day), but not when water (20 L) was added at three-day intervals [50]. The addition of copper did not affect the removal of COD, turbidity, or solids [50]. Obviously, any addition of copper or other biocidal material should occur near the outlet of the filter, as the dissolution of copper ions may result in the inactivation of the biomass that is responsible for the mineralization of carbon within the filter. Other media amendments such as bauxite and bentonite clay have been evaluated. Adding bentonite clay resulted in a significant improvement in bacteria retention within the filter bed [51]. The addition of small amounts of bentonite clay has been proposed to increase the surface area of adhesion sites for bacterial attachment and growth, thereby enhancing contaminant removal [52]. Slow sand filter amendments for arsenic removal (i.e., by retention within the filter) have been studied. Pre-oxidation using Fenton’s reagent and the subsequent removal of arsenic (As) through sand filtration from drinking water has also been proposed. The reported removal rate was 98 ± 2.5% [53]; however, long-term maintenance is expected to be an issue, as media replacement must eventually occur. Rather than adding amendments directly to the SSF, it may be more advisable to use a post-SSF “amendment-filter” to remove additional inorganic (i.e., metal or metalloid) contaminants.

#### 3.2.2. Media Depth

The recommended depth of sand for large units generally ranges between 1.2 and 1.4 m, although small household units have operated with sand depths of less than 0.3 m [22]. A minimum depth of 0.3 m is recommended for proper turbidity and coliform bacteria removal, and a depth of 0.6 m for significant virus removal [22]. However, it should be noted that the breakthrough of pathogenic bacteria and viruses cannot be completely eliminated even at these depths, and as long as turbidity and dissolved organics are effectively removed, post-filter disinfection is recommended.

Most organic carbon and microorganism removal in slow sand filters occurs near the top of the filter due to those processes mentioned above related to the development of the biofilm, creating a large microbial population within this zone. However, bed depth has also been shown to affect the removal efficiency (likely by affecting the HRT). Poynter and Slade (1977) recorded a slight decline in poliovirus removal from 99.98% to 99.94% when the filter media depth was reduced from 0.6 m to 0.3 m [15]. This bed depth dependency has not been universally reported, however, and even these numbers may not be statistically different. Bacterial removal has also been reported to be insensitive to variation in media depth. Coliform reduction declined by only 2% (from 97% to 95%) when sand depth was reduced from 0.97 m to 0.48 m [20,39]. This is consistent with the idea that bacterial removal occurs predominately in the first 30 cm of any slow sand filter. Again, the issue of depth versus any change in HRT needs to be noted.

### 3.3. Biological Maturity

Filter ripening over time significantly influences a filter’s microbial and organic contaminant removal rates [10,15]. The maturity period of slow sand filters is reported to vary from study to study and is likely a function of the influent water quality, HRT, and overall filter design. Reported maturation periods are 10–40 days [54] and 30 days [10]. A lab-based BSF operating for 27 days achieved a one log_10_ reduction in both fecal coliform and turbidity [55], while household BSF operation for 10 days achieved a 97% reduction in *E. coli*, total coliforms, and turbidity [56]. In summary, new filters require time for the microbial population, including in the Schmutzdecke layer, to grow and mature. Again, the influent water quality and consistency likely influence the rate of the initial microbial community growth.

Filter performance can be expected to continue to improve after the first month of operation due to incremental continued maturation, until clogging and/or preferential flow become an issue. Stauber et al. noted that *E. coli* reduction by household filters improved from 63% in unripe filters to 98 to 99% in fully mature filters [57]. For relatively new filters, the reduction in coliform bacteria and giardia were reported to be 85 and 98%, respectively, whereas in mature sand filters the reduction efficiencies were reported to be 99% and 100%, respectively [20,39]. These findings were supported by another study, which noted that the removal of coliform bacteria increased from 95% to over 99% as the filter matured and that the giardia removal rate was 98% in new filters, whereas in biologically mature filters, the removal rate was 3 to 4 logs [8,39]. Poynter and Slade (1977) reported that filter maturity improved bacteriophage reduction (99.45% vs. 99.99%) and poliovirus reduction (99.7% vs. 99.98%) [15]. While most reports suggest that the removal of bacteria and many other contaminants is influenced by the age of the filter [17], some researchers have reported no significant impact of filter maturity on contaminant removal. For example, Fogel et al. (1991) reported no observable effects on the removal of giardia cysts, cryptosporidium oocysts, turbidity, total coliforms, or fecal coliform bacteria due to the age of the biofilm [23].

After a filter is disturbed through cleaning (or through the inadvertent introduction of a microbial toxin or filter inactivity), it needs to reestablish a viable biological community. After cleaning, one study recorded a 17-day recovery period was necessary to achieve a one log_10_ reduction in fecal coliform [55]. This period was shorter than the 27-day maturity period for new filters for the same level of reduction [55]. Another study reported that cleaning or replacing filter media resulted in an initial one log_10_ decrease in bacteria removal efficiency [39]. Hence, while the frequency of cleaning is often measured in years, reduced performance can be expected to occur over the month immediately following such an event.

## 4. Operating Conditions

### 4.1. Hydraulic Residence Time

As stressed in the Introduction, filter efficiency (as measured by the percent removal of contaminants or the reduction in turbidity) is dependent on the contact time between the water and the filter media, i.e., the HRT. The HRT (h) is a function of the flow rate (Q, m^3^/h), total volume of the sand (V, m^3^), and sand porosity (*n*), and is mathematically defined by: HRT = V·*n*/Q. HRT is the key point of divergence between SSFs and rapid sand filters. For SSFs, Huisman and Wood (1974) suggested a Darcy flux of 0.1 to 0.4 m^3^/(m^2^·h) [14], though without explicitly stating the associated filter depth. Assuming a typical filter depth of 1 m and porosity of 0.5, the estimated hydraulic retention time for a slow sand filter would be 1.25 to 5 h. However considering the suggested 1 to 1.5 m of standing water above the sand layer [14], the HRT ranges from 2.5 to 12.5 h. The HRT rather than the Darcy flux is the most logical parameter for “sizing” an SSF. This is also because modern slow sand filters are constructed at different depths. Typically, community-scale filters may have a sand depth of 0.7 to 1.2 m, whereas filters designed for home use may have a depth as small as 0.2 m. Additionally, in batch-operated filters, the Darcy flux has no significance, as the water in these filters flows intermittently.

The HRT can be adjusted by varying (i) the hydraulic loading rate (L/day) in continuous-flow filters; (ii) the frequency of water addition (i.e., pause period) or water addition volume in intermittent filters; or (iii) the volume and/or porosity of the filter medium. As the HRT decreases (i.e., increase in hydraulic loading rate), a reduction in contaminant removal generally occurs. Logan et al. (2001) reported that the loading rate had a significant effect on the cryptosporidium cyst concentration in the effluent only when a coarse-grained medium was used [41]. Virus removal declined from 99.98% to 99.88%, with an increase in the Darcy flux (i.e., hydraulic loading rate) from 4.8 to 12 m/d [15]. A decrease in the hydraulic loading rate from 9.6 to 0.96 m/d was also reported to improve the removal of total coliform (98.98% vs. 99.96%), fecal coliform (98.65% vs. 99.84%), and turbidity (27.24% vs. 39.18%), respectively, but not the larger giardia cyst (99.991% vs. 99.991%) [20]. In this and other studies, viruses [39], cryptosporidium cysts [41], giardia [20], turbidity [20], coliform bacteria [20], and color [58] were reported to have increased the removal efficiency when filters were operated at higher hydraulic retention times.

For intermittent-flow filters, reducing the batch addition volume results in a longer overall residence time and often leads to an improved removal efficiency [40]. However, it must be noted that for this type of filter operation, there is an important relationship between the batch addition volume (V_b_) and the filter pore water volume (V_f_), where the filter pore water volume is equal to the total sand volume multiplied by the sand porosity (V_f_ = V·*n* = HRT·Q). Clearly, any batch water addition (V_b_) should be less than the filter pore volume (V_f_); otherwise, some of the water that is added will have an extremely short HRT. Obviously, if V_b_ > V_f_, some of the recently added water will undergo suspended solids removal, but very little removal of dissolved organic compounds will occur. Setting the value of V_b_ to 80% of the value of V_f_ is a reasonable approach (accounting for some dispersion), calculating V_f_ from the known value of V and an approximation of *n* (≈0.45). Using this approach, the volume of batch water addition, V_b_, becomes a set value for any given filter design, and the HRT is adjusted by varying the pause period between these batch water additions.

In a lab-scale study using filters with an estimated pore volume of 19.1 L, increasing the daily batch addition volume (V_b_) from 20 L to 40 L each day resulted in a log_10_ reduction in *E. coli* removal [59]. An earlier study also evaluated the effect of doubling the daily charge volume (from 20 L to 40 L) using household biosand filters with similar pore volumes (V_f_ = 19.1 L), reporting a reduction in bacterial removal. For this study, doubling the addition was achieved by adding the second 20 L an hour after the initial 20 L addition [45]. In both these studies, the doubled addition volume (V_b_ = 40 L) was more than the estimated filters’ water pore volume (V_f_ = 19.1 L), with approximately half of the water fed to the filters having a treatment time in the order of minutes.

In a 2011 study in which a PVC column was used as the filter, applying a longer overall residence time (i.e., 15.6 h vs. 5.1 h, on average) resulted in the improved removal of fecal coliform (1.59 vs. 1.30 log_10_), bacteriophage MS2 (0.72 vs. 0.38 log_10_), and turbidity (91.4 vs. 86.7%) [40]. A recent study confirmed this finding by reporting improved ammonia removal (71. 2 vs. 20.4) with a longer residence time (6 vs. 1 day(s)) [60]. Hyde et al. (2013) reported that household filters charged (i.e., batch fed) every 72 h demonstrated an improved coliform removal rate compared to those charged daily, with the same batch charge volume [50]. Tundia et al. (2016) observed improved microbial water quality with an increase in the length of the pause time from 12 to 36 h (at a constant V_b_) and a decrease in charge volume from 30 to 10 L (at a constant pause time). For their 19.6 L pore volume filters, Tundia et al. recommended a 23 h pause time between 19.4 L additions [61]. Thus, longer pause times [62], less frequent feeding, and smaller charge volumes generally result in better quality water. However, there is an optimum residence time that yields the best removal rate, as extended residence times can limit the growth and survival of the microbial community within a filter.

In another study, the outlets of SSFs were modified to determine the effect of the hydraulic retention time on the removal rates. The modified SSFs had a smaller outlet (from 0.5 inch to 0.37 or 0.25 inch). The intermittently operated filters were all operated with the same daily volume (V_b_ = 40 L). The study showed that no significant difference was observed in the outlet fecal coliform concentration between the modified filters (91.9–93.3%) and the control SSF (89.6%) [33].

Several studies have reported that (incremental, i.e., 2X) changes in residence time did not impact filter performance in continuous-flow filters. For example, Fogel et al. (1991) reported no observable effects on the removal efficiencies of cryptosporidium, giardia cysts, turbidity, and coliform bacteria over the flow-rate (i.e., Darcy flux) range of 0.19–0.4 m/h [23]. A separate pilot study further showed that even at loading rates of 0.4 m/h, the removal efficiency for giardia and coliform bacteria remained at over 99% [20]. For batch filters, a study reported that while maintaining V_b_ at a constant value, increasing the pause time from 12 to 72 h did not significantly affect the removal of turbidity, COD, or solids [50]. This was consistent with the idea that an HRT of 8 to 12 h is generally sufficient.

### 4.2. Temperature

Consistent with data showing lower contaminant removal rates at lower temperatures, the influence of temperature, in general, is related to its effects on microorganism metabolism [15]. For example, in mature filters, the removal efficiency of coliform bacteria was reported to be 97% at 17 °C and only 87% at 5 °C. While temperature was reported to have no significant effect on giardia removal, a reduction in temperature from 17 °C to 2 °C resulted in a 100-fold increase in the effluent coliform concentration [20,39]. In contrast, a pilot study demonstrated that virus reduction decreased from 99.999% (i.e., 5 log) to 99.8% (i.e., 2.7 log) when the temperature was reduced from 12 °C to 6 °C [15].

Some studies have concluded that the effect of temperature (under the specific operational conditions) was not significant. Fogel et al. (1991), for instance, reported no observable effects on the removal efficiency over the temperature range of 0.5 °C to 20 °C [23]. A separate study also reported no significant changes in the removal efficiency with short-term temperature reductions from 15 °C to 5 °C [63]. Again, whether a filter is operated at the margins of peak performance (i.e., HRT at or below 2 h) or under conditions where changes in other parameters are less influential on overall performance (i.e., HRT > 12 h) makes a difference to the importance of other control factors.

### 4.3. Mode of operation and O_2_ Requirements

Given the same HRT and filter design, the mode of operation—batch vs. continuous flow—also influences the filter performance. Although the smaller size of typical batch (i.e., intermittent-flow) filters does not by itself affect performance, the water in batch units does not continuously undergo dispersion, as occurs in continuous-flow filters. As a result, if not adsorbed directly onto the microorganism-coated sand during the initial batch water addition period, any dissolved organic material in the water will require more time to diffuse, through Brownian motion, to the medium surface, where the concentration of microorganisms is the largest, and where conversion to CO_2_ generally occurs. Indeed, Young-Rojanschi (2014) demonstrated that biosand filters were more efficient at removing *E. coli* (3.71 log_10_ vs. 1.67 log_10_), MS2 bacteriophages (2.25 log_10_ vs. 0.85 log_10_), and turbidity (96% vs. 87%) under continuous operation than under intermittent operation [64].

A simple modification to batch filters to potentially enhance performance would be the addition of a small water pump to recirculate water through the filter during the pause period, effectively inducing advective-dispersive flow during the pause period. This modification would also address an issue encountered when treating poor-quality water that presents high influent soluble carbonaceous and nitrogenous BOD (biochemical oxygen demand). Such a situation often occurs when attempting to treat household gray water. By recirculating water during the pause period, the filter has an additional HRT defined by the recycle flow rate, with this second HRT being much shorter that the overall HRT that is defined by the batch addition pause period.

### 4.4. Source Water Quality

The quality of the source water can affect the filter performance. A study reported that an increase in influent turbidity reduced MS2 removal in an SSF by 0.017–0.019 logs per NTU [40]. Influent turbidity also affects the removal efficiency of viruses [40]. In comparison, another study reported that while microbial loading in the feed water only marginally affected the removal of *E. coli* and coliform bacteria, a loading of 10^6^ MPN/L resulted in an 85% removal rate for both [17]. A Kenyan study of 30 households using in-home BSFs reported lower level of effluent fecal coliform in homes (a) that used rainwater only, (b) where children aged 6 to 10 did not collect the filtered water, or (c) that housed fewer family members [65]. The last factor was likely due to the over-use of the filters in homes with larger families, essentially decreasing the HRT, whereas the first factor was directly related to the influent water quality. A separate study reported that increasing nitrate loading from 27.1 to 32.5 g/m^2^ each day resulted in a decrease in the NO_3_^−^ removal efficiency from 99% to 94% [66]. Another study reported that using SSFs to treat rainwater achieved the required chemical and microbiological water quality standards, with cost savings of up to 60% when compared to the local water company [67].

Some studies have reported that for some source water quality parameters, filter performance was not affected by specific changes in these parameters. Fogel et al. (1991) reported that influent turbidity, total coliforms, and fecal coliforms did not affect the removal efficiency of protozoa [23]. Similarly, Tundia et al. (2014) reported that effluent microbial water quality was not impacted by variations in influent turbidity over the range of 10 to 50 NTU [61].

## 5. Contaminant Removal

Slow sand filtration has been effectively used to remove pathogenic microorganisms and other suspended organic and inorganic contaminants [8,15]. Studies have reported turbidity removal rates from 99 to 99.9% [39], bacteria removal rates from 99–99.9% [8], and virus removal rates of 2 to 6 logs [8]. A summary of the removal efficiency based on 18 water quality parameters reported in nearly 40 separate studies is provided in Table 2.

### 5.1. Protozoa and Larger Organisms

Slow sand filters have been reported to remove protozoa, such as cryptosporidium and giardia, and other pathogenic organisms from drinking water, as summarized in Table 2. One such organism is the larval form of Schistosoma (flatworm), known as the cercarial stage. Once ingested, the cercariae can develop into adult flatworms, which are responsible for the waterborne disease schistosomiasis. Kawata (1982) demonstrated the removal of cercariae using a PVC column sand filter, achieving 88.2 to 100% removal of the cercariae larva [70]. Using an acrylic filter, Pereira et al. achieved 97% removal of phytoplankton in a filter operated over a 70 day period [69].

The multiple log_10_ reduction in cryptosporidium by SSFs has long been established. Hijnen et al. (2004) performed column lab experiments in which the 5–6 log_10_ removal of *cryptosporidium* cysts was achieved. In another study, they reported a 4.7 log_10_ reduction [19,68]. This same research group also concluded that spores of *Clostridium perfringens* (SCP) and the small-sized centric diatom (SSCD) could be used as surrogates for cryptosporidium removal, reporting 3.6 and 1.8 log_10_ reductions in these indicator organisms, respectively [68]. In another study, a bench-scale sand filter was loaded at filtration rates of up to 10 cm/day and achieved 3 to 4 log_10_ cryptosporidium reduction [39]. Filter maturity and quality and the uniformity of the filter medium have been reported to affect cryptosporidium cyst removal [23,42].

High removal rates for giardia cysts by SSFs has been documented. As early as 1985, Bellamy et al. (1985) reported giardia removal rates ranging from 98% to 100% in a pilot-scale filter system [20,39]. Soon after, Schuler et al. (1991) reported giardia cyst removal rates of up to 100% in mature SSFs (over 99% in immature filters) with proper management [42]. A working (non-lab-based) filter achieved an average reduction of 93% for cysts found in a natural water source [23]. Thus, with proper management, slow sand filters are effective in reducing the concentration of giardia cysts in water.

### 5.2. Bacteria

As already mentioned, bacteria removal by SSFs has been well-documented. Pathogenic bacteria such as *Campylobacter* have been reported to be reduced by 3.2 to 4 log_10_ by slow sand filtration [19]. Tellen et al. (2010) demonstrated that the introduction of zerovalent iron into the sand improved the removal of total fecal Streptococci (99% vs. 95 to 98.6%) in CAWST (Centre for Affordable Water and Sanitation Technology) biosand filters [46]. However, due to the pathogenic nature of these and other bacteria, surrogate fecal bacteria (or classes of bacteria) such as total coliforms, fecal coliforms, and *E. coli* bacteria have been used in many studies [19]. Table 3 summarizes studies on bacteria removal by SSFs.

Coliform bacteria are any Gram-negative non-spore-forming rod-shaped bacteria that can ferment lactose, producing acid and gas at 35 °C. Fecal coliforms are facultative coliform bacteria that are capable of growing in the presence of bile salts and can produce acid and gas from lactose at 44 °C. *Escherichia coli (E. coli)* is a species of fecal coliform bacteria; however, it is not a dominant species by number in the intestines of humans, and it is completely absent in about 5% of the population. Slow sand filtration has been demonstrated to remove coliform bacteria, but often not to regulatory limits. Several researchers have published studies on coliform reduction via SSFs. The reported total coliform reductions include: 96% to 100% reduction [39], 85 to 99% removal in a plastic-bucket-based biosand filter [38], over 90% reduction [42], and 97% reduction by a household sand filter (HSF) in Pakistan [56].

Coliform removal has been reported to be influenced by several factors [35,39], such as temperature, source water quality [35], media quality, media amendments [46,50,71], sand size, residence time [50,61], and biological maturity [17,43,56]. Because most bacteria are removed in the top biofilm layer, removal rate has been reported to be rather insensitive to filter depth, as a reduction in medium depth from 0.97 m to 0.48 m resulted in a decline from 97% to 95% removal of coliform bacteria [8]. Uniform-sized clean (i.e., water-rinsed) sand and appropriate amendments are generally associated with better coliform removal. It appears that at least 1 or 2 log_10_ improvements in coliform removal are possible for mature filters. Coliform removal efficiency has also been reported to differ between field and lab-based filters. A Kenyan study on point-of-use concrete biosand filters reported slightly better removal rates for coliform in the lab when compared to field use (1.30 vs. 1.25 log_10_ reduction) [72]. This variation could be attributed to the better-controlled conditions in the lab, including the hydraulic residence time.

As an important indicator organism, the removal rates of *E. coli* during slow sand filtration have been studied extensively. The reported removal efficiencies are: 93% reduction in a 0.6 m deep lab-scale filter at a loading rate of 0.3 m/h [15], a 97% geometric mean reduction in rural Ghana [73], 96.3 to 97.9% mean reductions in Pakistan [56], and a 1.3 to 3.7 log_10_ reduction in household biosand filters [47].

The removal of E. coli is affected by the media quality [15]; media amendments [45,50,71]; mode of operation [15]; HRT [50,61]; filter maturity [17]; and study setting (lab, pilot, or field) [19,57]. In general, higher removal efficiencies for *E. coli* have been reported in laboratory (i.e., research)-operated filters compared to field-operated filters. A Dominican Republic study on household biosand filters reported mean *E. coli* reductions of 94% and 93% in lab and field studies, respectively [57]. A pilot plant study reported the removal of *E. coli* at a rate of 2 to 3 log_10_, whereas a study using field-based filters reported generally improved removal rates of 1.5 to 3.7 log _10_ [19]. A high removal efficiency variance (0 to 97%) has also been attributed to maturity, with removal rate increasing alongside duration of use [17].

### 5.3. Virus

While most studies have used bacteriophages as surrogates for disease-causing viruses, some studies have reported on actual removal efficiencies for pathogenic viruses. A lab-scale study reported poliovirus reductions of 98.25 to 99.997% [15]. Another study achieved a similar (99.9%) poliovirus removal rate with a filter bed depth of 0.6 m and a loading rate of 0.2 m/h [15]. A pilot study determined the removal efficiency of enteric viruses (poliovirus) to be higher than 1.8 log_10_ [54]. Since virus removal occurs primarily by microbial activity, filter maturity is critical [74]. Recent research has demonstrated that even with limited initial virus removal, a removal rate of up to 4 log_10_ can be achieved after a year of filter operation [74]. The reported removal efficiencies for viruses and their surrogates are summarized in Table 4.

Several studies have documented rotavirus removal in slow sand filters. Elliot et al. (2011), for instance, determined that the removal rate of rotavirus (PRD) was 0.053 log_10_ per hour (of pause time) for an intermittently operated household filter with an 18 to 20 h pause time [21]. In comparison, Yahya et al. (1993) reported a 99.9% removal rate for PRD through slow sand filters [75]. Wang et al. (2016) achieved a rotavirus reduction of 1.2 to 5.36 log_10_ using CAWST biofilters to treat groundwater [76]. Media amendment with iron filings has been reported to improve PRD removal (5.2 log_10_ vs. 1.1 log_10_) [34].

Bacteriophages are viruses that infect bacteria. Although bacteria and protozoa removal by biosand filters differs substantially depending upon the specific agent used [10], the removal of bacteriophages (MS2) has been reported to be comparable to that of viruses [15,19]. Therefore, these bacteriophages act as surrogates for virus removal. Reported phage removal rates include: 0.54 log_10_ MS2 average removal using a PVC column as the filter and 0.94 log_10_ MS2 average removal under optimum conditions [40], 99.75% to 99.996% reduction in bacteriophages at a loading rate of 0.3 m/h with a sand depth of 0.6 m [15], 99% removal of MS2 by an SSF [75], and 2.25 to 3.92 log_10_ removal of naturally occurring (native) somatic phages [54]. In pilot studies, Bauer et al. (2011) and Hijnen et al. (2004) determined bacteriophage removal rates of 12.5 to 4.01 log_10_ [54] and 1.5 to 2 log_10_ [19], respectively. Wang et al. (2014) estimated the reduction in MS2 bacteriophages to be between 4 to 7 log_10_ in CAWST concrete biosand filters with an average hydraulic residence time of 24 h [18]. Finally, a slow sand filter that used in a multistage filtration system achieved a 0.2 to 2.2 log_10_ reduction in MS2 phages [77].

As with bacterial removal, the removal of bacteriophages is affected by the filter characteristics and operating conditions [62]. Studies have reported that the continuous operation of household filters achieved higher bacteriophage removal rates than intermittent operation (2.25 log_10_ vs. 0.85 log_10_) [64]; media amendment with iron filings improved MS2 reduction (1 to 2 log_10_ vs. 3–4 log_10_) [34]; and spiking the source water with 1 mM NaHCO_3_ (sodium bicarbonate) impeded the removal of MS2 (1.2 log_10_ vs. 5 log_10_) [76].

### 5.4. Turbidity

Turbidity, caused by biological material and other suspended solids, is significant with respect to both the biological purity and the aesthetics of water [78]. While turbidity itself is not a direct health risk, it very often strongly correlates with the presence of pathogenic microorganisms [78] and can severely hinder secondary treatment with disinfectants. While the removal of turbidity from water does not ensure the removal of pathogenic organisms to regulatory limits, it does enable the application of low-cost disinfection methods, such as chlorination, ultraviolet (UV) light disinfection, and ozonation, to the effluent stream to obtain water that meets regulatory limits. Thus, turbidity removal is critical, as all these disinfection methods are less effective in water with higher turbidity. Fogel et al. (1991) demonstrated the relationship between effluent turbidity and bacterial removal, reporting that in over 90% of water samples with a turbidity of less than 1 NTU, no detectable total or fecal coliforms were present [23].

Water treated with slow sand filters often meets the 1 NTU limit, with exceptions most often occurring when the influent water is poor-quality surface water [42]. Reported effluent turbidity levels from household filters in actual home use include: 1.3 NTU [57], 0.6 to 1.5 NTU [56], 0.9 to 1.1 NTU [55], 0.9 NTU [35], less than 2 NTU in 95% of samples [69], and less than 1.5 NTU [61]. These published studies demonstrate that SSFs often achieve local regulatory turbidity limits.

Reported turbidity removal efficiencies range from as low as 27% to over 97%, as shown in Table 5. These studies reported: 97% ten-day removal in a household sand filter [56], 80 to 95% removal by a Kanchan^TM^ Arsenic Filter (KAF) [38], 94.5% removal of coliforms in contaminated raw waters [35], 55% average removal by an operational SSF [23], and 87 to 91% removal by a 60 L plastic sand filter. A randomized controlled trial in rural communities in Tamale, Ghana, reported a 67% geometric mean reduction in turbidity using biosand filters [73]. A six-month study involving 30 Kenyan households each using in-home biosand filters reported a 32% reduction in turbidity [65]. The wide variation in the removal of turbidity is associated with filter characteristics (including media characteristics); operating conditions (including HRT); and, most notably, source water quality, including the specific type of particles that are responsible for the turbidity.

Turbidity removal is influenced by the quality of the filter medium [71], medium amendments [45,46], HRT [40], filter maturity [17,56], and mode of operation [64]. The ability of a filter to remove turbidity also depends on the type of particles causing the turbidity. Inorganics, such as clay colloids, are often not effectively removed. A pilot study involving the treatment of lake water containing fine clay particles reported average turbidity removal rates in the range of 27 to 39% [20]. In our work in East Africa, we have encountered several surface and ground waters in Kenya and Tanzania containing high silicate colloid concentrations, particles which are also difficult to remove by slow sand filtration without a prior coagulation step. These examples indicate that turbidity does not always correlate to microbial contamination, as colloidal clays and silicates can cause high water turbidity with or without significant microbial contamination.

### 5.5. Organic Contaminants

We expect that over the next few decades, researchers will further examine the ability of slow sand filters to remove pharmaceutical and personal care products (PPCPs) [79], organic pesticides, and other micropollutants. Currently, in addition to monitoring the removal of turbidity (and visual observations of color), some studies have reported on organic matter removal (see Table 6). For example, it was reported that a batch-operated concrete BSF demonstrated an improved natural organic matter removal rate (54.0% vs. 47.6%) when the effective medium size was reduced from 0.3 to 0.15. mm [46]. A pilot-scale study demonstrated that slow sand filters exhibited the effective removal of biopolymers at temperatures above 15 °C and at biopolymer concentrations of less than 0.5 mg C/L [80].

### 5.6. Inorganic Contaminants

Slow sand filters, often with amendments or other modifications, have been shown to effectively remove certain inorganic contaminants, although it should be noted that most inorganic contaminants are recalcitrant (i.e., cannot be destroyed, such as As, Se, F, Cu, Co), and so filter modifications have been designed to retain one or more of these contaminants in the filter for a long period of time until the sorption capacity of the filter for the contaminant(s) is exceeded. The Kanchan^TM^ Arsenic Filter (KAF), a modified sand filter, removed 85 to 90% arsenic and 90 to 95% iron [38]. Nitrate, the most oxidized form of nitrogen, is likely to be removed by biofilms that have microanoxic environments, wherein the diffusion of molecular oxygen is limited and denitrification can occur, forming N_2_. In a study that measured nitrate removal by SSFs, the NO_3_-N removal efficiency was reported to be 94% [66]. In another study involving a layered (stratified) filter incorporating Bayer residue, zeolite, and fly ash, the filter achieved 97% removal of aluminum, 71% removal of TOC, and 88% removal of NH_4_ [44]. A separate study indicated that slow sand filters, through abiotic oxidation, biotic oxidation, precipitation, and sorption, could remove heavy metals such as manganese, iron, and arsenic [82]. A recent study supported these reported inorganic removal rates, citing 89% and 98% reductions in ammonium and manganese, respectively, using an optimized BSF [83]. The reports on inorganic contaminant removal by SSFs are summarized in Table 7.

## 6. Conclusions

For over two centuries, the application of SSFs for the treatment of municipal drinking water has been documented, with possible use occurring as far back as the Roman Empire, based on archeological evidence. Even with advancements in water treatment technology and knowledge, SSFs continue to be applied in the 21st century. There has, however, been a shift and expansion in use to smaller- and medium-scale systems, and SSF treatment is now often applied in combination with other water treatment processes, especially subsequent disinfection, with chlorination most often employed, as the complete removal of pathogens by SSFs is not guaranteed. Additionally, SSFs are now used for wastewater treatment (especially home greywater treatment and reuse) and industrial water treatment.

SSFs have been reported to remove several microbial, physical, and chemical contaminants. However, if subsequent disinfection is applied prior to water consumption, turbidity and organic (total organic carbon) removal becomes more important. In the case of chlorination, organic carbon in the water imparts a “chlorine demand” (due to reactions with this carbon, rather than with the target pathogens), such that more chlorine must be added to the water to overcome this demand, with the outcome being the formation of more disinfection by-products (DBPs). Several medium amendments have also been proposed to improve contaminant removal. However, we recommend that rather than adding amendments directly to the SSF, it may be more advisable to use a post-SSF “amendment filter” to remove additional inorganic (i.e., metal or metalloid) contaminants. SSFs are also not designed to treat water with a high mineral clay content, as these materials will clog the filter, requiring the more frequent complete removal and rinsing of the sand.

Several design and operational parameters affect SSF performance. Among these, the HRT and filter maturity are key. However, extended periods of time are generally not required for filters to be effective in reducing contamination, so the maturity period can be several days. As the HRT is vital to filter performance, pilot tests should be undertaken before major SSF design or implementation projects.

There are several research gaps in the SSF literature that need further study. More research and publications are required regarding SSF field deployment and maintenance. The literature on the costs (both capital and operational), maintenance, operation, and life-cycle analysis of SSF is limited. The published data are also predominantly from lab or pilot studies. The significance of microplastic contamination should also be explored, considering the recent spike in plastic BSFs.

## Figures and Tables

**Table 1:** Characteristics of slow sand filters.

Recommended by	Bed Depth (m)	Effective Medium Size (mm)	Darcy Filtration Rate (m/h)	Sand Uniformity Coefficient	Support Bed Depth (m)	Supernatant Water Depth (m)	Type of Filter
[14]	1.2	0.15–0.35	0.1–0.4	3.0		1–1.5	SSF
[26]	0.9	0.15–0.3	0.1–0.2		0.3–0.5	1	SSF
Ten States Standards USA (1987) [8]	0.8	0.3–0.45	0.08–0.24		0.4–0.6	0.9	SSF
[27,28]	0.6–1	0.1–0.3	0.1–0.3	<3			SSF
[29]	>0.5	0.15–0.2	0.4	1.5–2.5			Concrete BSF
[27]	>0.4	0.15–0.3	0.16–1.1	1.5–5			BSF

Adapted from Galvis et al. (2002) [30], Guchi (2015) [8], and Ngai and Baker (2014) [27].

**Table 2:** The removal of protozoa and larger organisms by slow sand filters.

Parameter	Setting	Filter Design			Operation		Removal Efficiency	Reference
		Material	Medium (Depth)	Dimensions/Area	Mode	Residence Time/Flow Rate		
Cryptosporidium parvum	Pilot		0.28 mm sand (1.5 m)	2.5 m^2^	Continuous	0.3 m/h	4.7 log_10_	[68]
	Pilot		0.3 mm sand	2.56 m^2^, depth = 1.5 m	Continuous	0.3 m/h	>5 log_10_	[19]
	Lab	PVC pipe	0.27 mm sand (0.9 m)	Diameter = 0.286 m	Continuous	0.15–0.4 m/h	>99.99%	[42]
	Field		0.2–0.3 mm sand (1.05 m)	43 × 6 m	Continuous	0.19–0.4 m/h	Average 48%	[23]
	Lab	Acrylic lysimeters	0.16 mm sand	Diameter = 0.15 m	Intermittent	0.04–0.1 m/h	>3 log_10_	[41]
Giardia cysts	Pilot	PVC pipe	0.29–0.62 mm sand (0.48–0.97 m)	Diameter = 0.3 m	Continuous	0.04–0.4 m/h	>98%	[20,39,63]
Phytoplankton	Lab	10 mm thick acrylic	0.18–2.83 sand (0.9 m)	0.2 × 0.2 m;	Continuous	1.8–7.1 m/day	Up to 97%	[69]
Schistome Cercariae	Lab	PVC pipe	0.1–0.4 mm sand (0.6–1.2 m)	Diameter = 0.152 m	Continuous	0.04–0.4 m/h	88.2–100%	[22,70]

**Table 3:** Bacteria removal by slow sand filters.

Parameter	Setting	Filter Design			Operation		Removal Efficiency	Reference
		Material	Medium (Depth)	Dimensions/Area	Mode	Residence Time/Flow Rate		
*E. coli*	Lab	Columns	0.17 mm sand (0.55 m)	Dia = 10 cm	Continuous	R. time = 1 day	3.71 log_10_	[64]
	Lab	Columns	0.17 mm sand (0.55 m)	Dia = 10 cm	Batch	R. time = 1 day	1.67 log_10_	
	Lab	Perspex cylinders	Sand (0.6 m)	0.09 m^2^, depth = 3 m	Continuous	0.2 m/h	>99.3%	[15]
	Pilot		0.3 mm sand	2.56 m^2^, depth = 1.5 m	Continuous	0.3 m/h	2–4.2 log_10_	[19]
	Field		0.13–0.6 mm sand	Length = 1–1.5 m	Continuous	0.25–0.4 m/h	1.5–3.7 log_10_	
	Field	Plastic filter	<1 mm sand (0.45 m)	60 L, depth = 0.76 m	Batch	Public use	0–97% (mean 93%)	[57]
	Lab	Plastic filter	<1 mm sand (0.45 m)	60 L, depth = 0.76 m	Batch	40 L/day	63–99% (mean 94%)	
	Lab	Plastic containers	0.23 mm sand (0.45 m)	p. vol. = 19.1 L, dia = 40 cm, depth = 73 cm	Batch	20–40 L/day	<1 log_10_–2 log_10_	[45]
	Lab	Plastic containers	0.23 mm sand (0.45 m) 10 cm iron-oxide-coated sand	p. vol. = 19.1 L, dia = 40 cm, depth = 73 cm	Batch	20–40 L/day	>2 log_10_ to >3 log_10_	
	Pilot	Concrete BSF	0.19 to 0.22 mm sand (0.45 m)	dia 15 cm, depth 90 cm	Batch	20 L/day	96.3–97.9%	[56]
	Lab	Plastic bucket	Sand (0.175 m)	25 L bucket, depth 41 cm	Batch	20 L/day	1.6–3.7 log_10_	[47]
	Lab	Plastic bucket	Sand (0.175 m) 10 cm of 3 mm zeolites	25 L bucket, depth 41 cm	Batch	20 L/day	1.3–3.7 log_10_	
	Lab	Concrete BSF	0.84–3.3 mm sand (0.44 m); 155 g chipped copper	Concrete BSFs (CAWST); 70 L	Batch	20 L/day	52–99.99%	[50]
	Lab	Concrete BSF	0.84–3.3 mm sand (0.44 m)	Concrete BSFs (CAWST); 70 L	Batch	20 L/day	20–99.99%	
	Lab	Concrete BSF	0.84–3.3 mm sand (0.44 m); 155 g chipped Cu	Concrete BSFs (CAWST); 70 L	Batch	20 L/3 day	45–99.99%	
	Lab	Concrete BSF	0.84–3.3 mm sand (0.44 m)	Concrete BSFs (CAWST); 70 L	Batch	20 L/3 day	22–99.99%	
	Lab	Concrete	0.18–0.3 mm sand	Concrete BSFs (CAWST) [29]	Batch	10 L/36 h–30 L/12 h	Optimum 99.4%	[61]
	Pilot	Polyethylene barrels	0.15–0.30 mm sand (0.57 m)	Dia 0.57 m, depth 0.88 m	Continuous	0.05 m/h	0–99.64%	[17]
	Field	Concrete	Sand (0.55 m)	Depth 95 cm, dia 35 cm, vol. 53.5 L		18 L/day	>95% by day 7	[43]
	Lab	Concrete	Contaminated sand (0.55 m)	Concrete BSFs (CAWST)		10 L/day	38 to over 99%	
	Lab	Concrete	Sand (0.55 m)	Concrete BSFs (CAWST)		10 L/day	71–99%	
	Lab	PVC columns	0.3–2.33 mm sand (0.45 m)	Depth 85 cm, dia 14.9 cm	Intermittent	8 L/12 h	97.8%	[71]
	Lab	PVC columns	0.3–2.33 mm sand (0.45 m); 21 g brass	Depth 85 cm, dia 14.9 cm	Intermittent	8 L/12 h	98.2%	
	Lab	PVC columns	0.3–2.33 mm sand (0.45 m); 39 g zerovalent iron	Depth 85 cm, dia 14.9 cm	Intermittent	8 L/12 h	97.33%	
Total Coliform	Lab	Perspex cylinders	Sand (0.6 m)	0.09 m^2^, d = 3 m	Continuous	4.8 m/day	>96.3%	[15]
	Pilot	PVC pipe	0.29–0.62 mm sand (0.48–0.97 m)	diameter = 0.3 m	Continuous	0.12 m/h	60.1–99.994%	[20,39]
	Field	Kanchan^TM^ Arsenic Filter (KAF)	Gravel, sand, and iron nails	Plastic or concrete bucket	Intermittent	Public use 10–15 L/h	60–99%	[38]
	Field	Concrete BSF	0.14 mm sand (0.5 m)	BushProof BSF: 0.95 m tall, 36 cm diameter	Intermittent	Public use	1.25 log_10_	[72]
	Field	Concrete BSF	0.14 mm sand (0.5 m)	BushProof BSF: 0.95 m tall, 36 cm diameter	Intermittent		1.30 log_10_	
	Lab	Concrete BSF	0.3 mm sand (0.5 m); 0.5 kg zerovalent iron	Concrete BSFs (CAWST)	Batch		99%	[46]
	Lab	Concrete BSF	0.3 mm sand (0.5 m)	Concrete BSFs (CAWST)	Batch		0–95%	
	Pilot	Concrete BSF	0.19 to 0.22 mm sand (0.45 m)	Dia 15 cm, depth 90 cm	Batch	20 L/day	97.2–97.4%	[56]
	Lab	10 mm thick acrylic	0.18–2.83 sand (0.9 m)	0.2 × 0.2 m	Continuous	1.8–7.1 m/day	50–99.98%	[69]
	Lab	Concrete BSF	0.84–3.3 mm sand (0.44 m); 155 g chipped copper	Concrete BSFs (CAWST); 70 L	Batch	20 L/day	33.3–99.8%	[50]
	Lab	Concrete BSF	0.84–3.3 mm sand (0.44 m)	Concrete BSFs (CAWST); 70 L	Batch	20 L/day	30–99.6%	
	Lab	Concrete BSF	0.84–3.3 mm sand (0.44 m); 155 g chipped Cu	Concrete BSFs (CAWST); 70 L	Batch	20 L/3 day	13.3–99.8%	
	Lab	Concrete BSF	0.84–3.3 mm sand (0.44 m)	Concrete BSFs (CAWST); 70 L	Batch	20 L/3 day	25.35–99.99%	
	Field	Concrete	Sand (0.55 m)	Depth 95 cm, dia 35 cm, vol. 53.5 L		18 L/day	>95% by day 7	[43]
	Lab	Concrete	Contaminated sand (0.55 m)	Concrete BSFs (CAWST)		10 L/day	64 to over 99%	
	Lab	Concrete	Sand (0.55 m)	Concrete BSFs (CAWST)		10 L/day	83–99%	
	Lab	Plastic bucket	As per CASWT specifications [29]; 0.14 mm sand (0.15 m)	30 L, dia 30 cm, depth 32 cm	Batch	20 L/12 h	Up to 1.5–2.2 log_10_	[35]
	Pilot	Polyethylene barrels	0.15–0.30 mm sand (0.57 m)	Dia 0.57 m, depth 0.88 m	Continuous	0.05 m/h	40.73–99.99%	[17]
	Lab	Concrete	0.18–0.3 mm sand	Concrete BSFs (CAWST) [29]	Batch	10 L/36 h–30 L/12 h	Optimum 99.7%	[61]
	Lab	PVC columns	0.3–2.33 mm sand (0.45 m)	Depth 85 cm, dia 14.9 cm	Intermittent	8 L/12 h	91.29%	[71]
	Lab	PVC columns	0.3–2.33 mm sand (0.45 m); 21 g brass	Depth 85 cm, dia 14.9 cm	Intermittent	8 L/12 h	90.11%	
	Lab	PVC columns	0.3–2.33 mm sand (0.45 m); 39 g zerovalent iron	Depth 85 cm, dia 14.9 cm	Intermittent	8 L/12 h	96.93%	
Fecal Coliform	Pilot	PVC pipe	0.29–0.62 mm sand (0.48–0.97 m)	Diameter = 0.3 m	Continuous	0.04–0.4 m/h	98.45–99.84%	[20,63]
	Lab	PVC	0.117–0.52 mm sand	Dia = 0.305 m	Batch	R. time 5–16 h (0.1–0.3 m/h)	1.4 log_10_	[40]
	Lab	Concrete BSF	0.3 mm sand (0.5 m); 0.5 kg zerovalent iron	Concrete BSFs (CAWST)	Batch		99%	[46]
	Lab	Concrete BSF	0.3 mm sand (0.5 m)	Concrete BSFs (CAWST)	Batch		0–95%	
	Lab	Plastic bucket	Sand (0.175 m)	25 L bucket, depth 41 cm	Batch	20 L/day	2.2–3.7 log_10_	[47]
	Lab	Plastic bucket	Sand (0.175 m); 10 cm of 3 mm zeolites	25 L bucket, depth 41 cm	Batch	20 L/day	2.7–4 log_10_	
	Lab	Plastic BSF	0.15–0.4 mm sand (0.43 m)	60 L BSF (HydrAid)	Batch	20 L/day	0.88–1.72 log_10_	[55]
	Field	Concrete BSFs (*n* = 155)	0.15 mm sand (0.5 m)	Depth 0.95 m, dia 36 cm	Batch	Public use 20 L/8 h	1.41 log_10_	[65]
	Lab	Plastic bucket	As per CASWT specifications [29]; 0.14 mm sand (0.15 m)	30 L, dia 30 cm, depth 32 cm	Batch	20 L/12 h	Up to 1.8–1.95 log_10_	[35]
Fecal Streptococci	Lab	Concrete BSF	0.3 mm sand (0.5 m); 0.5 kg zerovalent iron	Concrete BSFs (CAWST)	Batch		99%	[46]
	Lab	Concrete BSF	0.3 mm sand (0.5 m)	Concrete BSFs (CAWST)	Batch		0–95%	
Campylobacter	Field		0.13–0.6 mm sand	L = 1 to 1.5 m	Continuous	0.25–0.4 m/h	3.2–4.1 log_10_	[19]

**Table 4:** Virus and surrogate removal by slow sand filters.

Parameter	Setting	Filter Design			Operation		Removal Efficiency	Reference
		Material	Medium (Depth)	Dimensions/Area	Mode	Residence Time/Flow Rate		
Enteric Viruses (e.g., Polio Virus)	Lab	PVC pipe	0.27 mm sand (0.9 m)	Diameter = 0.286 m	Continuous	0.15–0.4 m/h	>99.83%	[42]
	Field		0.2–0.3 mm sand (1.05 m)	43 × 6 m	Continuous	0.19–0.4 m/h	Average 93%	[23]
	Pilot	Pond	(1.8 m) sample at 120 cm	3.25 × 6.85 m	Continuous	0.29 m/d	>1.8 log_10_	[54]
Bacteriophage (MS-2)	Lab	Perspex cylinders	Sand (0.6 m)	0.09 m^2^, depth = 3 m	Continuous	0.2 m/h	99.75–99.996%	[15]
	Pilot		0.3 mm sand	2.56 m^2^, depth = 1.5 m	Continuous	0.3 m/h	2.1 ± 0.6 log_10_	[19]
	Field	Plastic	Sand (4 ft)	5 × 10 ft, depth = 6 ft	Continuous		99%	[75]
	Pilot	Pond	(1.8 m) sample at 90 cm	3.25 × 6.85 m	Continuous	0.5–2 m/d	2.5–4.01 log_10_ (mean)	[54]
	Lab	Plastic BSF	0.15–0.4 mm sand (0.45 m)	60 L BSF (HydrAid)	Batch	20 L/day	1–2 log_10_	[34]
	Lab	Plastic BSF	0.15–0.4 mm sand (0.45 m); 5.54 kg mild iron filings	60 L BSF (HydrAid)	Batch	20 L/day	3–4 2 log_10_	
	Lab	Concrete BSF	Sand	Concrete BSFs (CAWST)	Batch	20 L/day	Average = 2 log_10_	
	Lab	Concrete BSF	Sand; 5.54 kg zerovalent iron	Concrete BSFs (CAWST)	Batch	20 L/day	7 log_10_	
	Lab	Concrete BSF	Sand; 0.26 kg steel wool	Concrete BSFs (CAWST)	Batch	20 L/day	>5 log_10_	
	Lab	Columns	224.1 g sand; 10% iron	Dia = 2.5 cm, vol = 146.7–161.5 cm^3^	Batch	R. time = 1 day	>5 log_10_ (mean)	
	Lab	Columns	224.1 g sand	Dia = 2.5 cm, vol = 146.7–161.5 cm^3^	Continuous	1.36 mL/min	1 log_10_ (mean)	
	Lab	Columns	0.27 mm sand (0.52 m)	Dia = 4.4 cm	Batch	450 mL/day	Up to 0.061 log_10_ per hour	[21]
	Lab	Columns	0.17 mm sand (0.55 m)	Dia = 10 cm	Continuous	R. time = 1 day	2.25 log_10_	[64]
	Lab	Columns	0.17 mm sand (0.55 m)	Dia = 10 cm	Batch	R. time = 1 day	0.85 log_10_	
	Lab	Concrete BSF	Sand (0.55 m)	Concrete BSFs (CAWST)	Batch	R. time 24 ± 8.5 h	4–7 log_10_	[18]
	Lab	PVC pipe	0.35 mm sand (0.55 m)	Dia = 4 inch	Intermittent	R. time 20–29 h	Average 5 log_10_	[76]
	Lab	PVC column	0.35 mm sand (0.4–0.9 m)	MS Filter Inc	Continuous	0.1–0.4 m/h	0.2–2.2 log_10_	[77]
	Lab	PVC	0.117–0.52 mm sand	Dia = 0.305 m	Batch	R. time 5–16 h (0.1–0.3 m/h)	0.54 log_10_	[40]
Rotavirus surrogate	Lab	PVC pipe	0.35 mm sand (0.55 m)	Dia = 4 inch	Intermittent	R. time 20–29 h	1.2–5.36 log_10_	[76]
	Field	Plastic	Sand (4 ft)	5 × 10 ft, depth = 6 ft	Continuous		99.9%	[75]
	Lab	Columns	224.1 g sand; 10% iron	Dia = 2.5 cm, vol = 146.7–161.5 cm^3^	Batch	R. time = 1 day	5.2 log_10_ (mean)	[34]
	Lab	Columns	224.1 g sand	Dia = 2.5 cm, vol = 146.7–161.5 cm^3^	Continuous	1.36 mL/min	1.1 log_10_ (mean)	
	Lab	Columns	0.27 mm sand (0.52 m)	Dia = 4.4 cm	Batch	450 mL/day	Up to 0.053 log_10_ per hour	[21]

**Table 5:** Turbidity removal by slow sand filters.

Setting	Filter Design			Operation		Removal Efficiency	Reference
	Material	Medium (Depth)	Dimensions/Area	Mode	Residence Time/Flow Rate		
Field		0.2–0.3 mm sand (1.05 m)	43 × 6 m	Continuous	0.19–0.4 m/h	Average 55%, effluent < 1.1 NTU	[23]
Lab	Columns	0.17 mm sand (0.55 m)	Dia = 10 cm	Continuous	R. time = 1 day	2.25 log_10_	[64]
Lab	Columns	0.17 mm sand (0.55 m)	Dia = 10 cm	Batch	R. time = 1 day	0.85 log_10_	
Lab	PVC	0.117–0.52 mm sand	Dia = 0.305 m	Batch	R. time 5–16 h (0.1–0.3 m/h)	89%	[40]
Field	Plastic filter	<1 mm sand (0.45 m)	60 L, depth = 0.76 m	Batch	Public use	Mean effluent = 1.3 NTU	[57]
Lab	Concrete BSF	0.3 mm sand (0.5 m); 0.5 kg zerovalent iron	Concrete BSFs (CAWST)	Batch		91.5–95%	[46]
Lab	Concrete BSF	0.3 mm sand (0.5 m)	Concrete BSFs (CAWST)	Batch		50–70%	
Lab	Plastic containers	0.23 mm sand (0.45 m)	p. vol. = 19.1 L, dia = 40 cm, depth = 73 cm	Batch	20–40 L/day	93%, effluent = 1 NTU	[45]
Lab	Plastic containers	0.23 mm sand (0.45 m); 10 cm iron-oxide-coated sand	p. vol. = 19.1 L, dia = 40 cm, depth = 73 cm	Batch	20–40 L/day	92%, effluent = 1 NTU	
Pilot	Concrete BSF	0.19 to 0.22 mm sand (0.45 m)	dia 15 cm, depth 90 cm	Batch	20 L/day	96.2–96.6%, effluent = 0.6–1.5 NTU	[56]
Lab	Plastic bucket	Sand (0.175 m)	25 L bucket, depth 41 cm	Batch	20 L/day	80%	[47]
Lab	Plastic bucket	Sand (0.175 m); 10 cm of 3 mm zeolites	25 L bucket, depth 41 cm	Batch	20 L/day	93%	
Lab	10 mm thick acrylic	0.18–2.83 sand (0.9 m)	0.2 × 0.2 m	Continuous	1.8–7.1 m/day	Effluent > 2 NTU (in 95% of samples)	[69]
Field	Concrete BSFs (*n* = 155)	0.15 mm sand (0.5 m)	Depth 0.95 m, dia 36 cm	Batch	Public use 20 L/8 h	Average 32.5%	[65]
Lab	Plastic BSF	0.15–0.4 mm sand (0.43 m)	60 L BSF (HydrAid)	Batch	20 L/day	87–91%, effluent = 0.9–1.1 NTU (average)	[55]
Lab	Plastic bucket	As per CASWT specifications [29]; 0.14 mm sand (0.15 m)	30 L, dia 30 cm, depth 32 cm	Batch	20 L/12 h	94.5%, effluent average 0.9 NTU	[35]
Pilot	Polyethylene barrels	0.15–0.30 mm sand (0.57 m)	Dia 0.57 m, depth 0.88 m	Continuous	0.05 m/h	0–82.63%	[17]
Lab	Concrete	0.18–0.3 mm sand	Concrete BSFs (CAWST)[29]	Batch	10 L/36 h–30 L/12 h	Effluent < 1.5 NTU	[61]
Lab	PVC columns	0.3–2.33 mm sand (0.45 m)	Depth 85 cm, dia 14.9 cm	Intermittent	8 L/12 h	88.71%	[71]
Lab	PVC columns	0.3–2.33 mm sand (0.45 m); 21 g brass	Depth 85 cm, dia 14.9 cm	Intermittent	8 L/12 h	88.5%	
Lab	PVC columns	0.3–2.33 mm sand (0.45 m); 39 g zerovalent iron	Depth 85 cm, dia 14.9 cm	Intermittent	8 L/12 h	91.5%	

**Table 6:** Inorganic contaminant removal by slow sand filters.

Parameter	Setting	Filter Design			Operation		Removal Efficiency	Reference
		Material	Medium (Depth)	Dimensions/Area	Mode	Residence Time/Flow Rate		
Natural Organic Matter (NOM)	Lab	Concrete BSF	0.3 mm sand (0.5 m)	Concrete BSFs (CAWST)	Batch		Average 47.64%	[46]
	Lab	Concrete BSF	0.15 mm sand (0.5 m)	Concrete BSFs (CAWST)	Batch		Average 54.0%	[46]
COD			Sand (fine/coarse)				50–74%	[22,81]
	Lab	Filter column	Sand (0.3 m) and GAC (0.2 m)	Height 0.65 m,	Continuous	5–10 cm/h	65.8%	[79]
TOC		Layered filter	Bayer residue, zeolite, and fly ash		Continuous and intermittent		71%	[44]
	Lab	Filter column	Sand (0.3 m) and GAC (0.2 m)	Height 0.65 m	Continuous	5–10 cm/h	90.3%	[79]
PPCPs	Lab	Filter column	Sand (0.4 m) and GAC (0.1 m)	Height 0.65 m	Continuous	10 cm/h	90.3%	[79]

**Table 7:** Inorganic contaminant removal by slow sand filters.

Parameter	Setting	Filter Design			Operation		Removal Efficiency	Reference
		Material	Medium (Depth)	Dimensions/Area	Mode	Residence Time/Flow Rate		
Nitrate	Lab	Stainless steel	0.5 mm sand (0.8 m)	Depth = 1 m, diameter = 0.1 m	Continuous	0.015–0.06 m/h	94–100%	[66]
NH_4_		Layered filter	Bayer residue, zeolite, and fly ash		Continuous and intermittent		88%	[44]
Arsenic	Field	Kanchan^TM^ Arsenic Filter (KAF)	Gravel, sand, and iron nails	Plastic or concrete bucket	Intermittent	Public use 10–15 L/h	88–95%	[38]
Iron	Field	Kanchan^TM^ Arsenic Filter (KAF)	Gravel, sand, and iron nails	Plastic or concrete bucket	Intermittent	Public use 10–15 L/h	>93%	[38]
Aluminum		Layered filter	Bayer residue, zeolite, and fly ash		Continuous and intermittent		95%	[44]
	Lab	PVC column	1.2 mm and 6.7 mm sand	0.25 m dia	Continuous	6 L/min, and 9.45 h HRT	98%	[83]
Managnese	Lab	PVC column	1.2 mm and 6.7 mm sand	0.25 m dia	Continuous	6 L/min, and 9.45 h HRT	88%	[83]

## Data Availability

Not applicable.
